# Maize *STEM BENDING1* controls plant architecture through cell wall and auxin pathways

**DOI:** 10.1007/s44307-026-00136-5

**Published:** 2026-10-16

**Authors:** Shiya Zhao, Babar Farid, Xintong Liu, Liang Dong, Fang Yang

**Affiliations:** 1 Guangdong Provincial Key Laboratory of Plant Resources, School of Agriculture and Biotechnology, Sun Yat-Sen University, Shenzhen, 518107 China; 2 National Key Laboratory of Crop Genetic Improvement, Huazhong Agricultural University, Wuhan, 430070 China; 3 Institute of Plant Breeding and Biotechnology, MNS University of Agriculture, Multan, 60000 Pakistan

**Keywords:** *Zea mays L*, Stalk lodging resistance, Stem bending, Secondary cell wall

## Abstract

Crop yield is largely determined by plant architecture. Identifying the key genes responsible for maize architecture can help optimize plant architecture for high-density planting. Here, we characterized a dominant *stem bending* mutant (*Stb1*) in maize, which exhibited bent stems and curved leaves leading to a slanted architecture. Genetic mapping delimited *Stb1* to a 3.7 Mb interval on chromosome 1. Transcriptome analysis suggested that *Stb1* mutation impaired the molecular pathways related to cell wall components and environmental stimuli. Consistently, lignin and hemicellulose contents were altered between the convex side and concave side of *Stb1* stems. In addition, the shoot gravitropism regulator *LAZY1* transcripts and endogenous auxin levels were differentially accumulated between the two sides of *Stb1* stems. Our study reveals that STB1 plays a role in maintaining upright stems through cell wall and auxin-related pathways, providing a new target for optimizing maize shoot architecture.

**Supplementary Information:**

The online version contains supplementary material available at https://doi.org/10.1007/s44307-026-00136-5.

## Introduction

Maize (*Zea mays L*.), as one of the most vital cereal crops worldwide, plays a crucial role in ensuring food security by serving as a major source of human food and animal feed. Driven by the pursuit of higher yield through increasing planting density accompanied by thinner and taller stems, stalk lodging has emerged as a major threat to sustainable maize production (Cui et al. 2022). To address this constraint, improving stem mechanical strength can not only effectively prevent yield loss from stalk lodging but also enhance maize tolerance to high-density planting. Stem mechanical bending strength is primarily determined by the cell wall structure components containing cellulose and lignin, and also the spatial morphology of stem, including stalk diameter, rind thickness (Robertson et al. 2017).

Recent studies have identified several key genes regulating stem mechanical rigidity and stalk lodging resistance in maize. For instance, the F-box domain protein *STIFF1* underlying a major stalk-strength QTL increases the cellulose and lignin accumulation in rind sclerenchyma cells of stem through activating the NAC-MYB expression (Zhang et al. 2020). Another stalk lodging major QTL, *stiff2*, which corresponds to the flowering-time gene *ZmCCT*, significantly enhances stalk bending strength by indirectly regulating a set of downstream genes involved in stalk elongation, stalk thickening, and lignin biosynthesis (Zhong et al. 2026). One class III peroxidase gene *ZmPRX38* acts downstream of the phenylpropanoid pathway to catalyze monolignol oxidation, and its favorable haplotype significantly improves stalk mechanical strength and yield performance (Wang et al. 2026). Identification of key genes for stem rigidity provide useful genetic resources for stem lodging resistance improvement. For example, targeted genetic modifications of cellulose synthase (*CESA*) genes and lignin pathways can effectively improve stem bending strength (Polko and Kieber 2019). Stem bending is also connected with shoot gravitropism. *LAZY1* (*LA1*) acts as a conserved master regulator of shoot gravitropism across rice, maize, and *Arabidopsis*, to control tiller/stem upright architecture through auxin signaling and gradient regulation (Li et al. 2007; Dong et al. 2013; Yoshihara et al. 2007). Mutating maize *la1* disrupts polar auxin transport (PAT) and distribution, leading to altered growth orientations and consequent stem lodging (Dong et al. 2013). However, while the roles of cell wall rigidity on stem lodging resistance have been documented, its molecular basis remains poorly understood. New genetic loci modulating stalk growth would expand our understanding on the mechanisms of stem strength and provide new targets for enhancing crop lodging resistance.

Here, we identify a natural maize mutant, *Stem bending 1* (*Stb1*), which displayed distinct stem bending, reduced plant height and curved leaves post-floral transition. Our results demonstrate that the *Stb1* locus is associated with altered stem cell wall structure and asymmetric auxin distribution, thereby providing new insights into the genetic basis of slanted plant architecture.

## Materials and methods

### Plant materials and phenotypic analysis

The dominant maize *Stb1* mutant was identified from natural variations in the B104 background. F_2_ mapping populations were generated by crossing *Stb1* (in B104) with the inbred line KN5585. Agronomic traits and yield components were evaluated using 10–20 plants per genotype.

### Histological observation

The 3rd to 4th stem internodes and ear leaves from WT and *Stb1* plants were fixed in FAA, embedded in paraffin and sectioned. Sections were stained with Toluidine Blue O (TBO) and imaged using a Nikon ECLIPSE Ni-U microscope. Cell size (average area of ≥ 60 cells across six independent microscopic fields of view from three biological replicates) and stomatal density were quantified using ImageJ. Cell number was enumerated per 1 mm^2^ area.

### Genetic analysis and BSR-seq

Genetic segregation in the F_2_ population (*n* = 245) was validated using the chi-square (χ^2^ test, df = 1) goodness-of-fit test. For Bulk Segregant RNA-seq (BSR-seq), equal amounts of total RNA from leaves of 20 extreme mutants and 20 WT F_2_ individuals were pooled. Sequencing reads were aligned using TopHat2 (Kim et al. 2013), and the locus was initially mapped via DeepBSA (Li et al. 2022). Fine mapping was performed using 12 polymorphic InDel and KASP markers (Supplemental Table 1) designed within the target region on chromosome 1 (252.8–289.2 Mb) using TBtools (Chen et al. 2020).

### Transcriptome and RT-qPCR analyses

Stem tissue was collected from approximately 30-day-old seedlings, with two biological replicates, and immediately frozen in liquid nitrogen. Total RNA was extracted using RNA extraction kit (Genestonebio, TR205, China), and libraries were constructed using Illumina Standard Library Preparation Kit. Each library generated approximately 20 M raw reads. RNA-seq reads from the 3rd to 4th internodes of WT and *Stb1* stems were mapped to the B73 reference genome (B73ref_v4) using TopHat2 (Kim et al. 2013). Differentially expressed genes (DEGs) were identified using DESeq2 (Love et al. 2014). To ensure statistical rigor, DEGs were filtered based on two key metrics: Log_2_ Fold Change (Log_2_FC), which quantifies the magnitude of expression ratio changes between *Stb1* and WT (with |Log_2_FC|> 1 indicating a greater than twofold difference in expression), and the False Discovery Rate (FDR), which adjusts raw *p*-values to control for false positives in multiple hypothesis testing using the Benjamini–Hochberg method (FDR ≤ 0.05). The identified DEGs were subsequently subjected to Gene Ontology (GO) enrichment analysis via TBtools (Chen et al. 2020). Relative expression of *ZmLA1* was quantified by RT-qPCR using elongation factor 1α as the internal control with three biological replicates (primers listed in Supplemental Table 2).

## Results

**Fig. 1:**
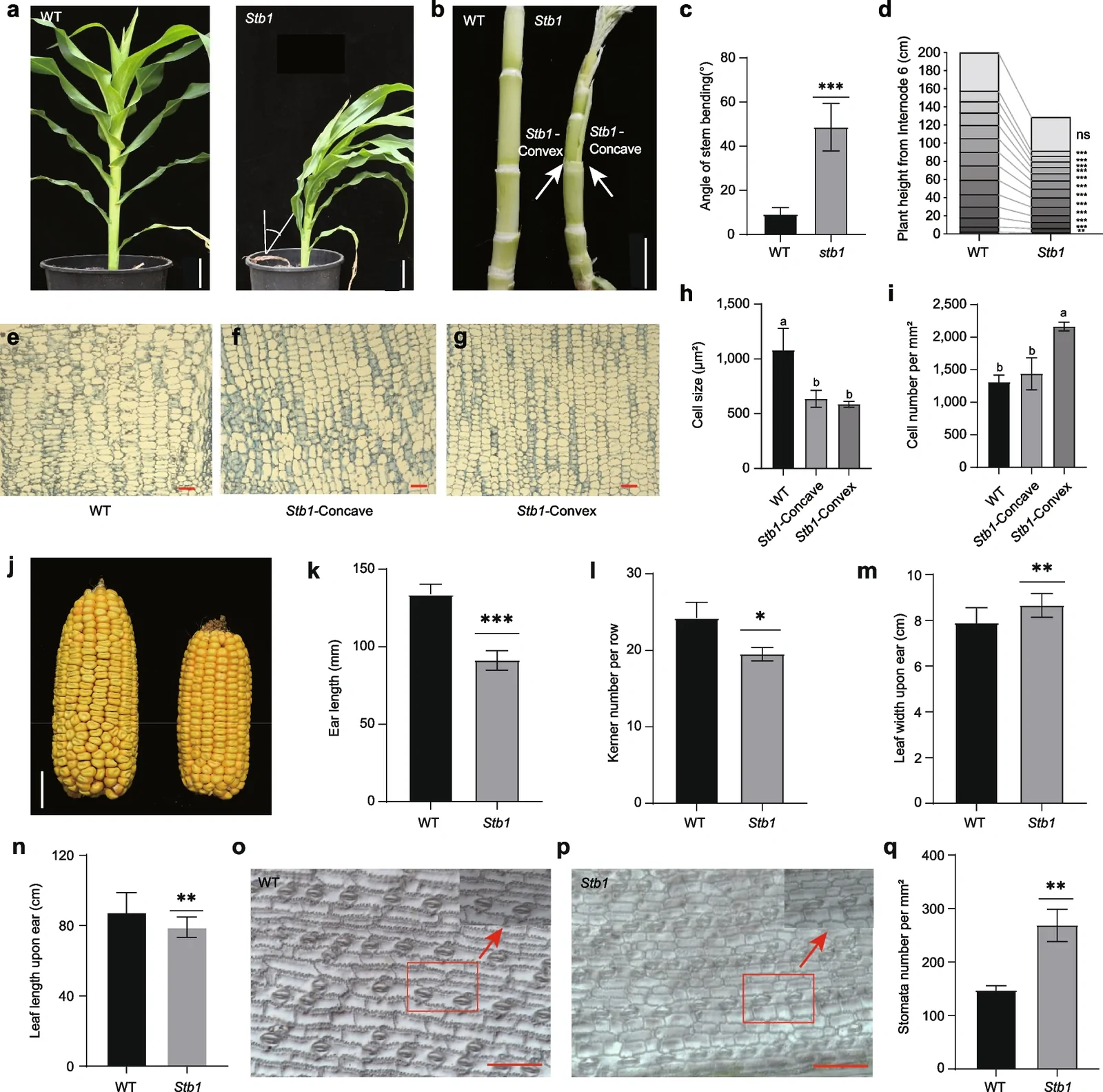
Phenotypic and cellular characterization of the *Stb1* mutant. **a** Gross morphology of wild-type (WT) and *Stb1* plants in the field. Bar = 10 cm. **b** Stem bending phenotype, with arrows indicating the inner concave side (*Stb1*-Concave) and outer convex side (*Stb1*-Convex). Scale bar = 10 cm. **c** Stem bending angle of WT and *Stb1*. **d** Comparison of internode length between WT and *Stb1* in maize mature period. 6i-18i indicates the number of internodes above the ground from bottom to top. **e–g** Stem cell observation of WT (e), concave side of *Stb1* (**f**), convex side of *Stb1* (**g**). Scale bar = 50 μm. **h**, **i** Comparison of cell size (h) and cell density (number per mm^2^) (i) in stem longitudinal sections (*n* = 3 biological replicates). **j** Comparison of mature ear of WT and *Stb1*. Scale bar = 10 cm. **k** Ear length of WT and *Stb1*. **l** Number of grains in rows of WT and *Stb1*. **m**, **n** Leaf width (m) and leaf length (n) of the leaf immediately above the uppermost ear (*n* = 20). **o**, **p** Abaxial leaf epidermal cells of WT (o) and *Stb1* (p), showing cell shrinking in the mutant. Scale bar = 100 μm. **q** Stomatal density on the abaxial leaf surface of WT and *Stb1* (*n* = 3 biological replicates). Data are presented as mean ± SD. In (c, d, k-n, q), statistical significance was determined by Student's *t*-test (*p < 0.05, **p < 0.01, ***p < 0.001). In (h, i), different lowercase letters indicate significant differences (*p* < 0.05) determined by one-way ANOVA with Tukey's multiple comparisons test

A natural maize mutant, *Stem bending 1* (*Stb1*), isolated from the B104 inbred line, exhibited distinct stem bending (~42° vs. ~ 10° in WT) and curved leaves post-floral transition (Fig. 1a-c). *Stb1* plants displayed reduced plant and ear heights due to shorter internodes, though internode number remained unchanged (Fig. 1d). Cellular analysis of *Stb1* stems revealed that while cells on both concave and convex sides were significantly smaller than those of WT, cell sizes between the two sides were comparable (Fig. 1e-h). Notably, cell number per unit area on the convex side was significantly higher than that on the concave side (Fig. 1i), suggesting that the accelerated cell division on the convex side could drive the differential stem growth and the subsequent bending. Additionally, *Stb1* mutants developed smaller ears with a ~ 32% reduction in ear length and a ~ 20% decrease in kernel number per row (KNPR) (Fig. 1j-l). The leaves above ear in *Stb1* were shorter and wider, displaying a ~ 9.67% decrease in length and a ~ 9.28% increase in width compared to WT (Fig. 1m, n). Cellular investigation revealed that the stomata number per unit area on the abaxial side of *Stb1* leaves was significantly increased compared to WT (Fig. 1o-q). Thus, the *Stb1* mutation triggers pleiotropic developmental and agronomic phenotypes in maize.

**Fig. 2:**
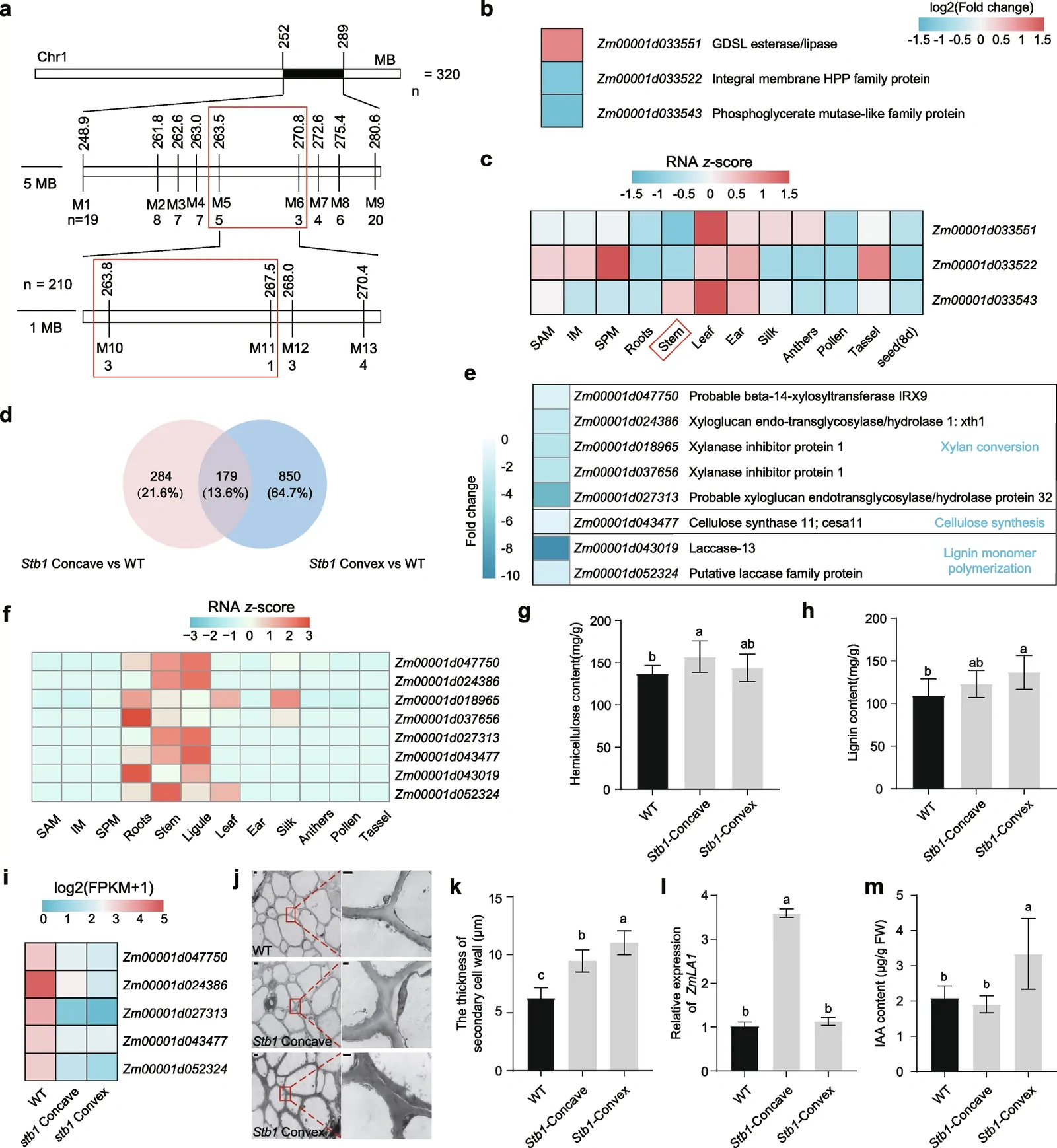
Fine mapping of *Stb1* and multi-dimensional analysis of downstream pathways. **a** Fine mapping of the *Stb1* locus on chromosome 1, narrowing the candidate interval to 3.7 Mb between markers M10 and M11 (n, number of F_2_ segregating individuals). **b** Expression changes (log_2_ fold change) of the three candidate genes within the localized genomic window. **c** Tissue-specific expression profiles of the three candidate genes based on public datasets.** d** Venn diagram showing overlapping and distinct DEGs in *Stb1*-Concave vs. WT and *Stb1*-Convex vs. WT. **e**, **f** Expression changes (e) and tissue-specific expression profiles (f) of key DEGs involved in xylan transferase, cellulose synthesis, and lignin polymerization. **g**, **h** Quantification of hemicellulose (g) and lignin (h) contents in the stems of WT, *Stb1*-Concave, and *Stb1*-Convex. **i** FPKM values of DEGs associated with cell wall composition in *Stb1* stems **j**, **k** Transmission electron microscopy (TEM) images (j) and quantification of secondary cell wall thickness (k) in WT, *Stb1*-Concave, and *Stb1*-Convex stem internodes (*n* = 3 biological replicates). Scale bars = 20 μm (left) and 5 μm. **l** Relative expression level of the *ZmLA1* gene across genotypes and stem sides. **m** Endogenous IAA content in the stems of WT, *Stb1*-Concave, and *Stb1*-Convex. *Stb1*-Concave and *Stb1*-Convex represent the inner concave and outer convex sides of the bent stem, respectively. Data are presented as mean ± SD. Different lowercase letters indicate significant differences (*p* < 0.05) determined by one-way ANOVA with Tukey's multiple comparisons test

To determine the genetic cause of the *Stb1* mutation, an F_2_ population was developed by crossing *Stb1* with KN5585 and further self-crossing. The F_2_ population segregated in a 3:1 (*Stb1*: WT) ratio, indicating that the stem bending phenotype was controlled by a single dominant locus (χ^2^ test, Fig. S1a). Bulk segregant RNA-seq (BSR-seq) initially mapped the *Stb1* mutation to a ~ 37 Mb region on chromosome 1 with an allele frequency difference of 0.99 between pools (Fig. S1b-d). Subsequent fine mapping with 320 F_2_ individuals narrowed the locus to a 7.3 Mb interval (Chr1: 263.5–270.8 Mb) between markers M5 and M6 (Fig. 2a, Fig. S2a, b). Utilizing polymorphic InDel markers M10 and M11 (Fig. S2c, d) on 210 segregating plants further refined the candidate region to a 3.7 Mb interval (Chr1: 263.8–267.5 Mb) containing 69 annotated genes (Fig. 2a, Supplemental Table 3). Among these, three genes were differentially expressed based on the stem transcriptome of *Stb1* (Fig. 2b). Notably, *Zm00001d033543* encoding a phosphoglycerate mutase exhibited high stem expression in public RNA-seq datasets (Fig. 2c) (Yi et al. 2019; Yu et al. 2022; Sun et al. 2024), identifying it as a prime *Stb1* candidate requiring further functional validation.

To explore the downstream pathways of *Stb1*, we conducted transcriptome analysis on bolting-stage stems (|Log_2_ FC|> 1, FDR ≤ 0.05). We identified 463 differentially expressed genes (DEGs) on the concave side (162 up-regulated and 301 down-regulated) and 1,029 DEGs on the convex side (619 up-regulated and 410 down-regulated) compared to WT (Fig. 2d; Fig. S3a-b). Additionally, 840 DEGs were identified between the concave and convex sides (585 up-regulated and 255 down-regulated) (Fig. S3c). Gene Ontology (GO) analysis revealed significant enrichment in environmental stimulus responses, cell differentiation, and cell wall metabolism/biogenesis (Fig. S3d-f). Notably, eight cell wall-related genes were downregulated in *Stb1* stems, including five xylan transferase, one cellulose synthase, and two lignin polymerization-related genes (Fig. 2e). Five of these genes (*Zm00001d047750*, *Zm00001d024386*, *Zm00001d027313*, *Zm00001d043477*, and *Zm00001d052324*) exhibited high stem-specific expression in public datasets (Fig. 2f), suggesting their potential roles in stem development. To evaluate cell wall properties, we quantified structural components in *Stb1* stems. Hemicellulose content was elevated overall, with the concave side containing significantly more hemicellulose than the convex side (Fig. 2g). Compared to WT, lignin content was significantly higher on the convex side (Fig. 2h), providing structural rigidity for the rapidly expanding tissue. This was consistent with the upregulated expression of xylan transferase genes on the concave side (Fig. 2i), whereas cellulose content remained unchanged (Fig. S4a). Microscopic analysis further revealed that *Stb1* developed significantly thicker secondary cell walls than WT (Fig. 2j, k). Collectively, these asymmetric alterations in cell wall composition and structure disrupt tissue mechanical balance, driving the stem bending phenotype.

Our transcriptome analysis revealed that *LAZY1* (*LA1*), a classical regulator of shoot gravitropism (Dong et al. 2013), was upregulated in *Stb1* stems. Real-time qPCR confirmed that *ZmLA1* transcripts were significantly more abundant on the concave side than those on the convex side (Fig. 2l). Given that *ZmLA1* regulates auxin pathway, we quantified endogenous auxin levels and found that IAA significantly accumulated at a higher level on the convex side of *Stb1* stems compared to those on the concave side and in the segregated WT (Fig. 2m). Consistently, the stem-preferential auxin-responsive gene *SAUR71* (*Zm00001d026308*) was upregulated in *Stb1*, showing more pronounced induction on the convex side (Fig. S4b). These results demonstrate that *Stb1* maintains upright stem growth by modulating *ZmLA1* expression and proper auxin gradients across opposing stem sides.

## Discussion

Stalk lodging represents a primary constraint to sustainable maize production under high-density cultivation. In this study, we characterized the stem-defective mutant *Stb1*, which displayed pleiotropic phenotypes, including stem bending, reduced plant height with shortened internodes, and smaller ears, etc. (Fig. 1). The asymmetric mechanical properties driving *Stb1* stem bending likely originate from cell wall compositional imbalance. Transcriptome analysis revealed widespread dysregulation of genes associated with cell wall metabolic processes (Fig. 2e), with significant alterations in cellulose synthases and xylan biosynthetic genes critical for secondary cell wall assembly. Biochemical quantification showed that hemicellulose content was significantly higher on the concave side, whereas lignin content was elevated on the convex side. Hemicellulose normally provides structural flexibility via cross-linking with cellulose microfibrils, while lignin acts as a hydrophobic filler that rigidifies the cell wall matrix, restricting cell expansion and flexibility (Mengistie and McDonald 2024). This unbalanced composition likely alters the local elastic properties of opposing stem sides, which may in turn contribute to bending.

Consistent with our observations, alterations in cell wall composition have been associated with stem bending phenotypes in several plant species. In *Arabidopsis*, overexpression of a pectin methylesterase inhibitor (PMEI) causes stems to develop twists and loops, accompanied by altered cell wall sugar composition and elasticity (Müller et al. 2013). In rice, *THE FLEXIBLE CULM 1* (*FC1*) gene encodes a cinnamyl-alcohol dehydrogenase (CAD), a key enzyme in the lignin biosynthesis pathway. Loss of *FC1* function leads to reduced lignin content and altered monomer composition, resulting in decreased cell wall thickness, which may compromise stem mechanical support and ultimately cause a flexible and bending stem phenotype (Li et al. 2009; Hirano et al. 2012). This observation echoes the asymmetric lignin deposition pattern on the convex and concave sides of *Stb1* stems, suggesting that alterations in cell wall components, particularly lignin metabolism, may contribute to the bending phenotype by affecting the local mechanical properties of opposing stem sides. Together, these observations suggest that changes in cell wall composition and structure may disrupt tissue mechanical balance and flexibility, thereby contributing to stem bending or morphological abnormalities across diverse plant species.

Asymmetric auxin distribution is a prerequisite for organ bending upon environmental stimuli (Rashotte et al. 2001). This process relies heavily on polar auxin transport (PAT) mediated by *LAZY* genes (Dong et al. 2013; Li et al. 2007). The localized upregulation of *ZmLA1* on the concave side of *Stb1* may impair lateral or basipetal PAT, potentially leading to hyper-accumulation of auxin on the convex side and contributing to the bending phenotype. Interestingly, cell wall integrity signaling and auxin have been shown to interact in regulating asymmetric growth (Walia et al. 2024). We propose that in *Stb1*, cell wall-derived mechanical cues and auxin-dependent growth signals may function in a coordinated manner to influence differential growth on opposing stem sides.

Although the *Stb1* locus has been successfully delimited to a 3.7 Mb candidate interval containing 69 annotated genes, identifying the exact functional candidate is the first focus of next work. The direct regulatory pathways of *STB1* will then be further investigated after the causal gene is isolated. Nevertheless, our findings establish *STB1* as a vital regulator of maize upright stem growth and shoot architecture. Elucidating the STB1-mediated coordination of cell wall properties and mechanical strength provides valuable insights into breeding lodging-resistant elite maize germplasm.

## Supplementary Information

Supplementary Material 1: Supplementary Fig. S1 | Genetic analysis and BSR-seq gene mapping. a Genetic analysis of* Stb1*. b BSR-seq to obtain the primary localisation interval on maize chromosome 1. c Initial positioning results of Deep learning algorithm. The abscissa represents the length of chromosome 1-10 of maize, and the ordinate represents the calculated value of the algorithm. d Initial localization interval on chromosome 1 in maize. Left, peak, and right indicate the corresponding positions within the localization interval, and value represents the correlation score. Supplementary Fig. S2 | KASP and InDel markers for gene mapping. M5 represents KASP marker. M6, M10 and M11 represent Indel markers. a M5: 263.5Mb KASP; b M6: 270.8 Mb; c M10: 263.8Mb; d M11: 267.5Mb. W represents wild type (KN5585), and M represents *Stb1* mutant (B104), F1 represents KN5585/B104. Supplementary Fig. S3 | Volcano plot of DEGs and GO enrichment in *Stb1* a-c Volcano plots displaying differentially expressed genes (DEGs) between (a) *Stb1* concave and WT (301 down-, 162 up-regulated), (b) *Stb1 *convex and WT (410 down-, 619 up-regulated), and (c) *Stb1* concave and *Stb1* convex (255 down-, 585 up-regulated). d-f GO enrichment analyses of DEGs for the respective comparisons in (a-c). Supplementary Fig. S4 | Cellulose content and expression profile of the auxin-responsive gene *SAUR71* in* Stb1* a Cellulose content in the stems of wild type (WT) and the concave and convex sides of *Stb1 *bending stems. Data are presented as mean ± SD. Different lowercase letters indicate statistically significant differences (*P*< 0.05). b Expression analysis of *SAUR71* (*Zm00001d026308*) in WT and the concave and convex sides of* Stb1 *stems based on RNA-seq profiling. c Tissue-specific expression pattern of *Zm00001d026308 *across various developmental tissues in maize (data adapted from Han et al. 2023). Color scale represents relative expression levels.

Supplementary Material 2.

## Data Availability

The RNA sequencing data generated in this study have been deposited in the NCBI BioProject (https://www.ncbi.nlm.nih.gov/bioproject/) under accession number PRJNA1522360. All other data that support the findings of this study are available from the corresponding authors on request.
