# Supplementary material for: Maize STEM BENDING1 controls plant architecture through cell wall and auxin pathways
Source: Adv Biotechnol (Singap). 2026 Oct 16;4(4):41. doi: 10.1007/s44307-026-00136-5 (PMC13675445; doi:10.1007/s44307-026-00136-5)
Supplement: Supplementary file 1 [file 44307_2026_136_MOESM1_ESM.docx]

**Maize *STEM BENDING1* controls plant architecture through cell wall and auxin pathways**

Shiya Zhao^1,2^, Babar Farid^3^, Xintong Liu^1^, Liang Dong^1^* and Fang Yang^1,2^*

^1^Guangdong Provincial Key Laboratory of Plant Resources, School of Agriculture and Biotechnology, Sun Yat-sen University, Shenzhen 518107, China

^2^National Key Laboratory of Crop Genetic Improvement, Huazhong Agricultural University, Wuhan 430070, China

^3^Institute of Plant Breeding and Biotechnology, MNS University of Agriculture, Multan 60000, Pakistan

^*^Corresponding author: yangf389@mail.sysu.edu.cn; dongliang7@mail.sysu.edu.cn

**Supplementary Fig. S1 | Genetic analysis and BSR-seq gene mapping.**

**a** Genetic analysis of *Stb1*. **b** BSR-seq to obtain the primary localisation interval on maize chromosome 1. **c** Initial positioning results of Deep learning algorithm. The abscissa represents the length of chromosome 1-10 of maize, and the ordinate represents the calculated value of the algorithm. **d** Initial localization interval on chromosome 1 in maize. Left, peak, and right indicate the cor-responding positions within the localization interval, and value represents the correlation score.

**Supplementary Fig. S2 | KASP and InDel markers for gene mapping.**

M5 represents KASP marker. M6, M10 and M11 represent Indel markers. a M5: 263.5Mb KASP; b M6: 270.8 Mb; c M10: 263.8Mb; d M11: 267.5Mb. W represents wild type (KN5585), and M represents *Stb1*mutant (B104), F1 represents KN5585/B104.

**Supplementary Fig. S3 | Volcano plot of DEGs and GO enrichment in *Stb1*.**

**a-c** Volcano plots displaying differentially expressed genes (DEGs) between (a) *Stb1* concave and WT (301 down-, 162 up-regulated), (b) *Stb1* convex and WT (410 down-, 619 up-regulated), and (c) *Stb1* concave and *Stb1* convex (255 down-, 585 up-regulated). **d-f** GO enrichment analyses of DEGs for the respective com-parisons in (a-c).

**Supplementary Fig. S4 | Cellulose content and expression profile of the auxin-responsive gene *SAUR71* in *Stb1*.**

**a** Cellulose content in the stems of wild type (WT) and the concave and convex sides of Stb1 bending stems. Data are presented as mean ± SD. Different lowercase letters indicate statistically significant differences (*P* < 0.05). **b** Expression analysis of *SAUR71* (*Zm00001d026308*) in WT and the concave and convex sides of *Stb1* stems based on RNA-seq profiling. **c** Tissue-specific expres-sion pattern of *Zm00001d026308* across various developmental tissues in maize (data adapted from Han et al. 2023). Color scale represents relative expression levels.
