# Supplementary material for: Maize STEM BENDING1 controls plant architecture through cell wall and auxin pathways
Source: Adv Biotechnol (Singap). 2026 Oct 16;4(4):41. doi: 10.1007/s44307-026-00136-5 (PMC13675445; doi:10.1007/s44307-026-00136-5)
Supplement: Supplementary file 2 [file 44307_2026_136_MOESM2_ESM.docx]

**Supplemental Table 1| Markers primers for fine mapping**

| **Primer name** | **Primer sequence** | **Locations** |
| --- | --- | --- |
| M1-F | CGATGAAGACAAGGCCAGTT | Chr1:248985782-248989824 |
| M1-R | CCACTGTTTTGACGGCCTAT |  |
| M2-F | TGAGTTGATCTGATCGCGGG | Chr1:261892154-261892554 |
| M2-R | TGCTTGTCACATCAGTAACCG |  |
| M3-F | GCAAGATGTTCTCCCGTGCT | Chr1:262685818-262690887 |
| M3-R | CGGCTCAAGCCTAACCCAT |  |
| M4-F | GCCCGTACCATAGAATCCGG | Chr1:263012444-263013873 |
| M4-R | CGAACGAGAAGCGAAGGACT |  |
| M5-F1 | GAAGGTGACCAAGTTCATGCTAGACCATGATTTGCAGTCTACTTCTA | Chr1:263524019-263524246 |
| M5-F2 | GAAGGTCGGAGTCAACGGATTAGACCATGATTTGCAGTCTACTTCTG |  |
| M5-R | CAGAGTCATATGCCAGAACGAAAC |  |
| M6-F | GAGGAACTTGGGGAGGTGTG | Chr1:270870843-270871443 |
| M6-R | GGGCTGAAATTTCGGGTTCG |  |
| M7-F | ACCCAGACGAAGGTGTTGAT | Chr1:272699913-272710052 |
| M7-R | TTGGTCTCATGTCGTCTGGA |  |
| M8-F | TCTGGTCCAGGCCTAGTGAT | Chr1:275417610-275425788 |
| M8-R | GCCATGCTTTCTCCTAAACA |  |
| M9-F | CATGCCAGCGTGGTTAAATA | Chr1:280602552-280608350 |
| M9-R | TGACATGTATGGAATGGACAGC |  |
| M10-F | GGCTGCAAAGTAAGCCTGTT | Chr1:263884476-263885008 |
| M10-R | ACACCGCTGTTGATCTTGTC |  |
| M11-F | GGAACCTGTGACGTGATGCC | Chr1:267546318-267547018 |
| M11-R | TTTACTAGGACGCACACACG |  |
| M12-F | CGGGAAAGGACACTTCAGGT | Chr1:268093040-268093840 |
| M12-R | AGAGTTGACTGCATGCCTCC |  |
| M13-F | ATAAACATGTAGGGCGGCCC | Chr1:270494465..270495265 |
| M13-R | CGGACACTGGCATTCCTGTA |  |

**Supplemental Table 2| RT-qPCR primers**

| Primer name | | Primer sequence |
| --- | --- | --- |
| *EF1*α-F | ACTGAGGTGAAGTCTGTGGAAATGC | |
| *EF1*α-R | TTTGGTGGGTCGTTCTTGCTGTC | |
| *ZmLA1*-F | GTGAAAGGGACGACAGGAAA | |
| *ZmLA1*-R | AGATCTGGAGGATCTTGTGA | |
